# Analysis of *Notch1* signaling in mammalian sperm development

**DOI:** 10.1186/s13104-023-06378-z

**Published:** 2023-06-19

**Authors:** Naoto Sambe, Masaharu Yoshihara, Teppei Nishino, Ryosuke Sugiura, Takahiro Nakayama, Chandra Louis, Satoru Takahashi

**Affiliations:** 1 College of Medicine, School of Medicine and Health Sciences, University of Tsukuba, 1-1-1 Tennodai, Tsukuba, Ibaraki, 305-8575 Japan; 2 PhD Program in Humanics, School of Integrative and Global Majors, University of Tsukuba, 1- 1-1 Tennodai, Tsukuba, Ibaraki, 305-8577 Japan; 3 Department of Primary Care and Medical Education, Institute of Medicine, University of Tsukuba, 1-1-1 Tennodai, Tsukuba, Ibaraki, 305-8575 Japan; 4 Department of Anatomy and Embryology, Institute of Medicine, University of Tsukuba, 1-1-1 Tennodai, Tsukuba, Ibaraki, 305-8575 Japan; 5 Department of Medical Education and Training, Tsukuba Medical Center Hospital, 1-3-1 Amakubo, Tsukuba, Ibaraki, 305-8558 Japan; 6 PhD Program in Human Biology, School of Integrative and Global Majors, University of Tsukuba, 1-1-1 Tennodai, Tsukuba, Ibaraki, 305-8577 Japan; 7 Laboratory Animal Resource Center in Transborder Medical Research Center, University of Tsukuba, 1-1-1 Tennodai, Tsukuba, Ibaraki, 305-8575 Japan

**Keywords:** Cre/loxP system, Delta-Notch signaling pathway, Gal4/UAS system, Single-cell RNA-seq, Transgenic mouse

## Abstract

**Objective:**

A mammalian Delta-Notch signaling component, *Notch1*, has been suggested for its expression during the normal sperm development although its conditional deletion caused no apparent abnormalities. Since we established our original transgenic mouse system that enabled labeling of past and ongoing *Notch1* signaling at a cellular level, we tried to validate that observation in vivo. Our transgenic mouse system used Cre/loxP system to induce tandem dsRed expression upon Notch1 signaling.

**Results:**

To our surprise, we were unable to observe tandem dsRed expression in the seminiferous tubules where the sperms developed. In addition, tandem dsRed expression was lacking in the somatic cells of the next generation in our transgenic mouse system, suggesting that sperms received no *Notch1* signaling during their development. To validate this result, we conducted re-analysis of four single-cell RNA-seq datasets from mouse and human testes and showed that *Notch1* expression was little in the sperm cell lineage. Collectively, our results posed a question into the involvement of *Notch1* in the normal sperm development although this observation may help the interpretation of the previous result that *Notch1* conditional deletion caused no apparent abnormalities in murine spermatogenesis.

**Supplementary Information:**

The online version contains supplementary material available at 10.1186/s13104-023-06378-z.

## Introduction

*Notch1* is a component of the mammalian Delta-Notch signaling pathway that consists of five Delta ligands (*Delta-like ligands 1*, *3*, and *4* and *Jagged 1* and *2*) and Notch receptors (*Notch 1*, *2*, *3* and *4*) [1]. Upon binding with Delta ligands, the Notch intracellular domain is cleaved, translocates to the nucleus, and acts as a transcription factor to induce specific genes. These genes include *Hes*/*Hey* family proteins that are expressed in many organs including inner ear and cerebellum [2, 3]. Although it has been suggested that the intracellular domains of *Notch1* and *Notch2* were similar and interchangeable with each other, the loss-of-function effect to organ development is different by the Notch receptor subtypes owing to the difference of their extracellular domains [4, 5]. Therefore, it should be important to distinguish the contribution of each Notch receptor by their subtypes.

Concerning gamete formation, this signaling pathway is involved in nematodes. For example, in *Caenorhabditis elegans*, genetic deletion of germline proliferation-1 (*GLP-1*) Notch receptor allowed all germline stem cells to enter the meiotic cell cycle, suggesting that this signaling pathway is necessary for maintenance of germline stem cells [6]. In *Drosophila melanogaster*, the numbers of germline cells or supporting cells were decreased by reduction of Delta ligand or Notch receptor expression, respectively [7]. In *Xenopus laevis*, migration of the primordial germ cells to the future gonad was disrupted by *X-Delta-2* knockdown [8].

The mammalian spermatogenesis involves multiple differentiation steps including spermatogonial cells (before meiosis), spermatocytes (during meiosis), spermatids (after meiosis) and mature sperm (spermatozoa) that are supported by the gonadal cells other than testicular germ cells such as Sertoli cells, Leydig cells and interstitial cells [9]. The involvement of *Notch1* in the murine spermatogenesis was studied by Huang et al. using the transgenic mice that enabled spermatocytes-specific gain-of-function and loss-of-function [10]. Concretely, since immunohistochemistry for *Notch1* suggested its expression in the wild-type mouse spermatocytes, they investigated the effect of spermatocyte-specific gain-of-function of *Notch1* by using *stra8-iCre* mouse and showed that that led to impaired spermatogenesis. In the same report, however, loss-of-function of *Notch1* in the spermatocytes caused no apparent dysfunction in spermatogenesis. Given that Huang et al. used no primary antibody negative control instead of the testis samples from *stra8-iCre*-mediated spermatocyte-specific *Notch1* conditional knockout mice, the contribution of *Notch1* to spermatogenesis in the wild type mice is still unclear. In addition, Hayashi et al. reported the presence or absence of *Notch1* in the spermatocytes of the rat testis or human patients with arrested spermatocyte maturation, respectively [11]. Owing to lack of knockout study in that report, however, it is still controversial whether the absence of *Notch1* in those human patients was the cause of the arrested spermatocyte maturation.

To examine the cell lineage receiving *Notch1* signaling in the mammalian testis, we combined experimental and bioinformatics methods. Recently, we established a transgenic mouse system that enabled visualization of past and ongoing *Notch1* signaling that consisted of three transgenic mouse lines. The first transgenic mouse expresses Notch1 receptor of which intracellular domain is replaced with a transcription factor, Gal4VP16 (*N1-Gal4VP16* mouse) [12]. The second transgenic mouse expresses Cre recombinase upon the binding of Gal4VP16 to its upstream activating sequences (UAS) (*UAS-Cre* mouse) [13]. The third transgenic mouse expresses EGFP or tandem dsRed (tdsRed) before and after Cre-mediated recombination, respectively (*R26GRR* mouse) [14]. Therefore, in *N1-Gal4VP16; UAS-Cre; R26GRR* mouse, past and ongoing *Notch1* signaling could be labeled with tdsRed or Cre recombinase, respectively. Importantly, since the transgenic mice used in the present study were the transgenic mouse in the narrowest sense (*N1-Gal4VP16* mouse and *UAS-Cre* mouse) or *ROSA26* locus-targeted knock-in mouse (*R26GRR* mouse), the expression levels of *Notch1* were assumed to be comparable to that of the wild-type mouse. Using this transgenic mouse system, we examined past and ongoing *Notch1* signaling in the murine sperm cell lineage.

Unfortunately, immunohistochemistry for Notch1 was technically difficult to us, we evaluated our result from the transgenic mouse experiments by re-analyzing four series of the previously published single-cell RNA-seq (scRNAseq) data (GSE104556 and GSE112393 from mouse testes) (GSE124263 and GSE142585 from human testes) [15–18].

## Main text

### Materials and methods

#### Animal Welfare

Animal experiments were carried out in accordance with the Regulation for Animal Experiments in our university and Fundamental Guidelines for Proper Conduct of Animal Experiment and Related Activities in Academic Research Institutions under the jurisdiction of the Ministry of Education, Culture, Sports, Science and Technology. Approval was obtained from the Institutional Animal Care and Use Committee and the DNA Experiment Committee of the University of Tsukuba (Approval Numbers for Animal Experiments: 22–059) (Approval Number for DNA Experiments: 220,018). *UAS-Cre* mice are available from RIKEN through the National BioResource Project of Japan: *Crl:CD1(ICR)-Tg(UAS-cre/T2A/miRFP670)216Staka* (No. RBRC11716). In addition, *R26GRR* mice are also available from RIKEN through the National BioResource Project of Japan: *C57BL/6 N-Gt(ROSA)26Sor < tm1(CAG-EGFP/tDsRed)Utr>/Rbrc* (No. RBRC04874).

#### Animal experiment

The post-weaning mice (4–23 weeks old) were used for histological (n = 3) and PCR (n = 6) examination. PCR templates (n = 6) were derived from a single *N1-Gal4VP16; UAS-Cre; R26GRR* father. When sampling the embryos at embryonic day 14.5 (E14.5), the pregnant mother was euthanized by cervical dislocation and the embryos were immediately fixed in ice-cold Mildform 10 N (Cat #: 133-10311, Wako, Osaka, Japan). PCR primers for the detection of *EGFP* coding region were 5’-AGCAAGGGCGAGGAGCTGTTCACC-3’ (forward) and 5’-TGCCGTCGTCCTTGAAGAAGATG-3’ (reverse). In prior to sampling the organs, mice were sacrificed by cervical dislocation and perfused with PBS and Mildform 10 N. For fluorescent imaging, 10 μm thick frozen sections were counterstained with Hoechst 33,342 (Cat #: H3570, Invitrogen, Waltham, MA, USA). For immunohistochemistry, 4 μm thick paraffin sections were incubated with or a rabbit monoclonal anti-Cre recombinase antibody (clone: D7L7L, Cat #: 15,036 S, RRID: AB_2798694, Cell Signaling Technology, Danvers, MA, USA), Histofine SimpleStain (Cat #: 414,341, Nichirei Biosciences, Tokyo, Japan), and Histofine DAB Substrate Kit (Cat #: 425,011, Nichirei Biosciences), followed by counterstaining with Mayer’s hematoxylin solution (Cat #: 131–09665, Wako). Images were captured by using BIOREVO-BZ-X810 (Keyence, Osaka, Japan).

#### ScRNA-seq analysis

We downloaded the four datasets of the previously published scRNAseq (GSE104556 and GSE112393 from mouse testes) (GSE124263 and GSE142585 from human testes) [15–18] from NCBI Gene Expression Omnibus (https://www.ncbi.nlm.nih.gov/geo/). We used Scanpy (v1.7.0) [19] to conduct quality control, dimension reduction with principal component analysis (“scanpy.tl.pca” function) and UMAP (“scanpy.tl.umap” function) before clustering (Leiden method or Louvain method). Single-cell RNA sequencing analysis was completed in Python 3.6.13 in an Ubuntu 20.04 LTS environment.

For the mouse dataset (GSE104556), we used 2524 cells and 2976 highly variable genes (the original dataset was provided after quality control). For the other mouse dataset (GSE112393), we used 33,468 cells and 5494 highly variable genes after removing the dead cells with more than nine mitochondrial gene counts per cell and removing the doublet cells judged by Scrublet (v0.2.3) [20]. For the human dataset (GSE124263), we selected and used 4561 cells out of 3065 highly variable genes after removing the doublet cells judged by Scrublet (v0.2.3) [20]. For the other human dataset (GSE142585), we used 13,628 cells and 6267 highly variable genes after removing the doublet cells judged by Scrublet (v0.2.3) [20]. The following marker genes were used: *Spag6* (Sperm) [21], *Prm1* (Sperm) [22], *Hspa1l* (spermatid, ST) [23], *Ccna1* (spermatocyte, SC) [24], *Stk31* (SC) [25], *Dmrt1* (spermatogonial cell, Spg) [26], *Csnk2a1* (Spg) [27], *Wt1* (Sertoli cell) [28], *Cyp17a1* (Leydig cell) [29], *Cd68* (macrophage, macro) [30], *Acta2* (interstitial cell, Int) [31], *Cd34* (Blood and vascular cell, BV) [32].

## Results

### Animal experiments

**Fig. 1:**
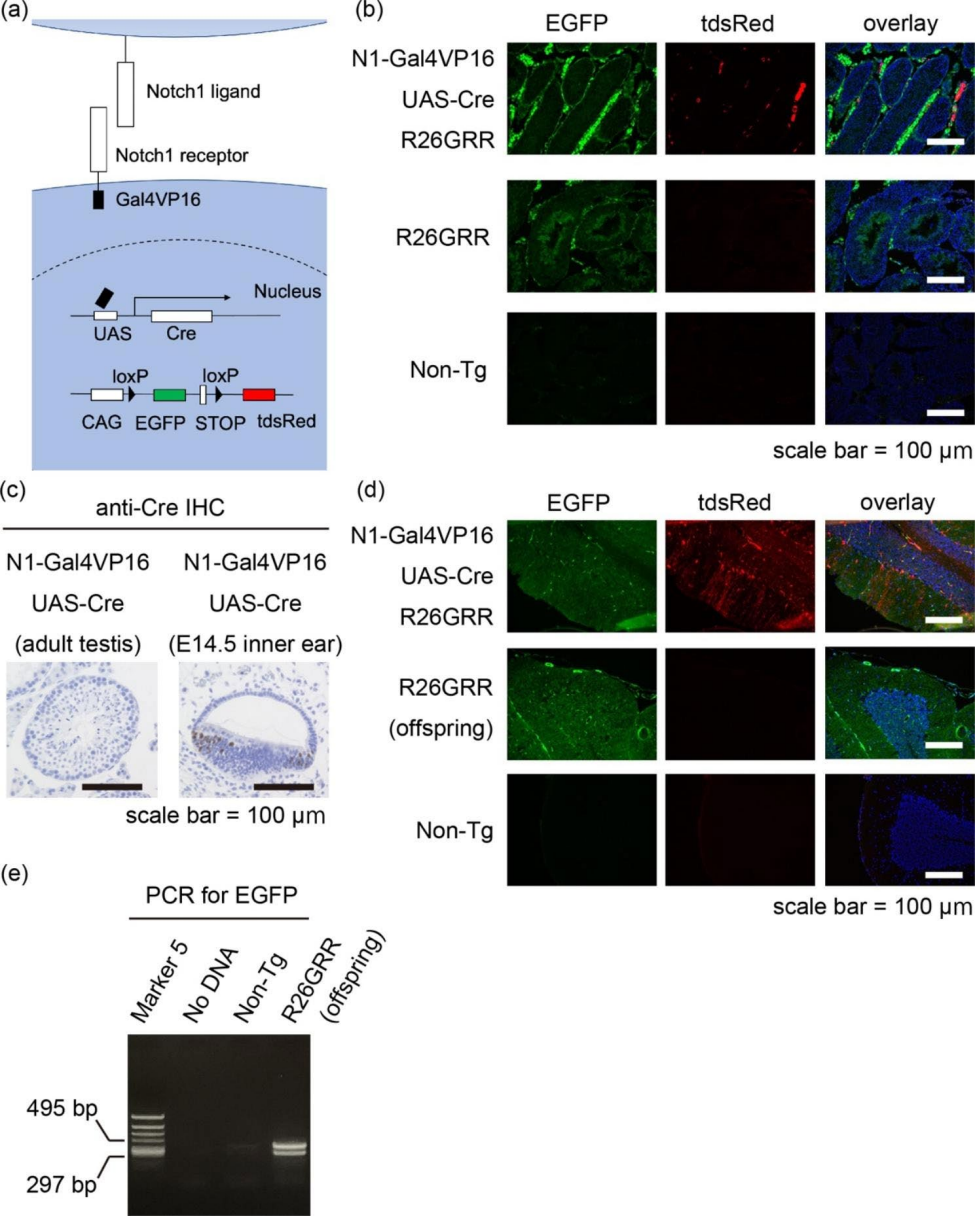
Transgenic mouse experiment revealed lack of *Notch1* signaling in the sperm. (**a**) Schematic representation of our transgenic mouse system. Note that *Notch1* signaling results in the expression of Cre recombinase (ongoing *Notch1* signaling) and tdsRed (past *Notch1* signaling). (**b**) Fluorescent examination of the seminiferous tubules. “Non-Tg” means, hereafter, the mice without any of “*N1-Gal4VP16*”, “*UAS-Cre*,” or “*R26GRR*” transgene elements. (**c**) Immunohistochemistry for Cre recombinase using the testes from adult *N1-Gal4VP16; UAS-Cre* mice. Right panel shows positive control (inner ear from an E14.5 *N1-Gal4VP16; UAS-Cre* mouse embryo). (**d**) Fluorescent examination of the cerebellum from mice of the indicated genotypes. (**e**) PCR for *EGFP* showed bands at the expected size (296 base pairs) in the *R26GRR* lane, suggesting that these cells developed devoid of Cre-mediated recombination.

Our transgenic mouse system was summarized in Fig. 1a. We started from the fluorescent observation of the seminiferous tubules (Fig. 1b). We observed tdsRed fluorescence (past *Notch1* signaling) in the stroma of *N1-Gal4VP16; UAS-Cre; R26GRR* mouse. Unfortunately, the EGFP or tdsRed fluorescence in the seminiferous tubules were dim (n = 3 from single-time observation), we carried out immunohistochemistry for Cre recombinase to examine ongoing *Notch1* signaling (Fig. 1c). Although some of the inner ear cells at E14.5 *N1-Gal4VP16; UAS-Cre* mouse embryo expressed Cre recombinase (positive control), we never observed Cre-expressing cells in the seminiferous tubules. Given this result, we hypothesized that the sperms developed independently of *Notch1* signaling. Since *Notch1* signaling in the developing sperms would result in tdsRed expression in the somatic cells of the next generation, we examined the cerebellum of the *R26GRR* offspring (Fig. 1d). As expected, tdsRed fluorescence was lacking in the *R26GRR* offspring in contrast to that of *N1-Gal4VP16; UAS-Cre; R26GRR* mouse (positive control). In addition, we detected PCR bands for *EGFP* using the tail DNA from the *R26GRR*-harboring offspring as the template (Fig. 1e). Full-length blots/gels are presented in Additional File 1. Collectively, these results suggested that the sperms developed independently of *Notch1* signaling.

### An scRNAseq analysis of mouse testes (GSE104556)

**Fig. 2:**
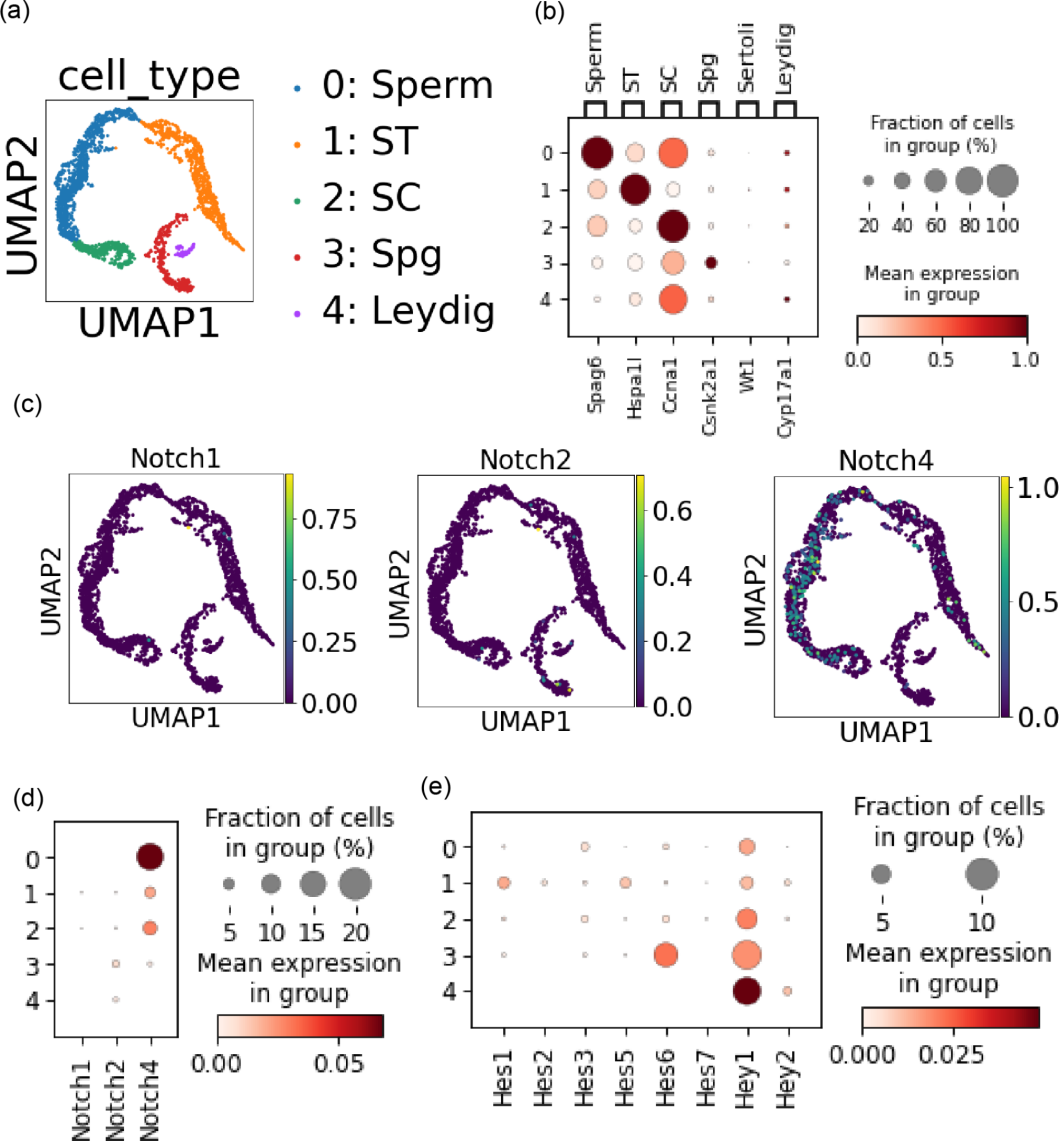
An scRNAseq analysis of mouse testes revealed little *Notch1* expression. (**a**) Five clusters from the mouse testes dataset (GSE104556) on UMAP. (**b**) Characterization of each cluster with indicated marker genes. (*c*) The expression levels of Notch receptors were plotted on the UMAP. (**d**) Dot plots for Notch receptor expressions. (**e**) The expression levels of the downstream targets of Notch signaling were plotted.

To examine our experimental findings, we next carried out scRNA-seq re-analyses of two previously published mouse testes datasets (GSE104556 and GSE112393) [15, 16]. To examine species difference, we also carried out scRNA-seq re-analyses of two previously published human testes datasets (GSE124263 and GSE142585 from human testes) [17, 18] in Additional File 2. The first mouse testes dataset (GSE104556) [15] was derived from two wild-type mouse. UMAP clustering yielded five clusters (Fig. 2a). We tried to characterize each cluster using established marker genes: cluster #0 as sperms, cluster #1 as spermatids (ST), cluster #2 as spermatocytes (SC), cluster #3 as spermatogonial cells (Spg), cluster #4 as Leydig cells (Fig. 2b). Then, we plotted the expression profile of Notch receptors (Fig. 2c). *Notch1* and *Notch2* were rarely expressed in each cluster whereas *Notch4* was observed in cluster #0 (sperm), #2 (SC), #1 (ST) at low expression levels (less than 0.1 per 10,000 mRNA counts) (Fig. 2c and d). *Notch3* expression was so little that it was not considered as a highly variable gene. Among *Hes*/*Hey* family downstream target genes, *Hey1* (cluster #4, Leydig cell) and *Hes6* (cluster #3, Spg) were expressed at most 10% of each cluster at very low expression levels (Fig. 2e). These results implied that, at least, *Notch1* was not the major receptor in the murine developing testicular germ cells.

### Another scRNAseq analysis of mouse testes (GSE112393)

**Fig. 3:**
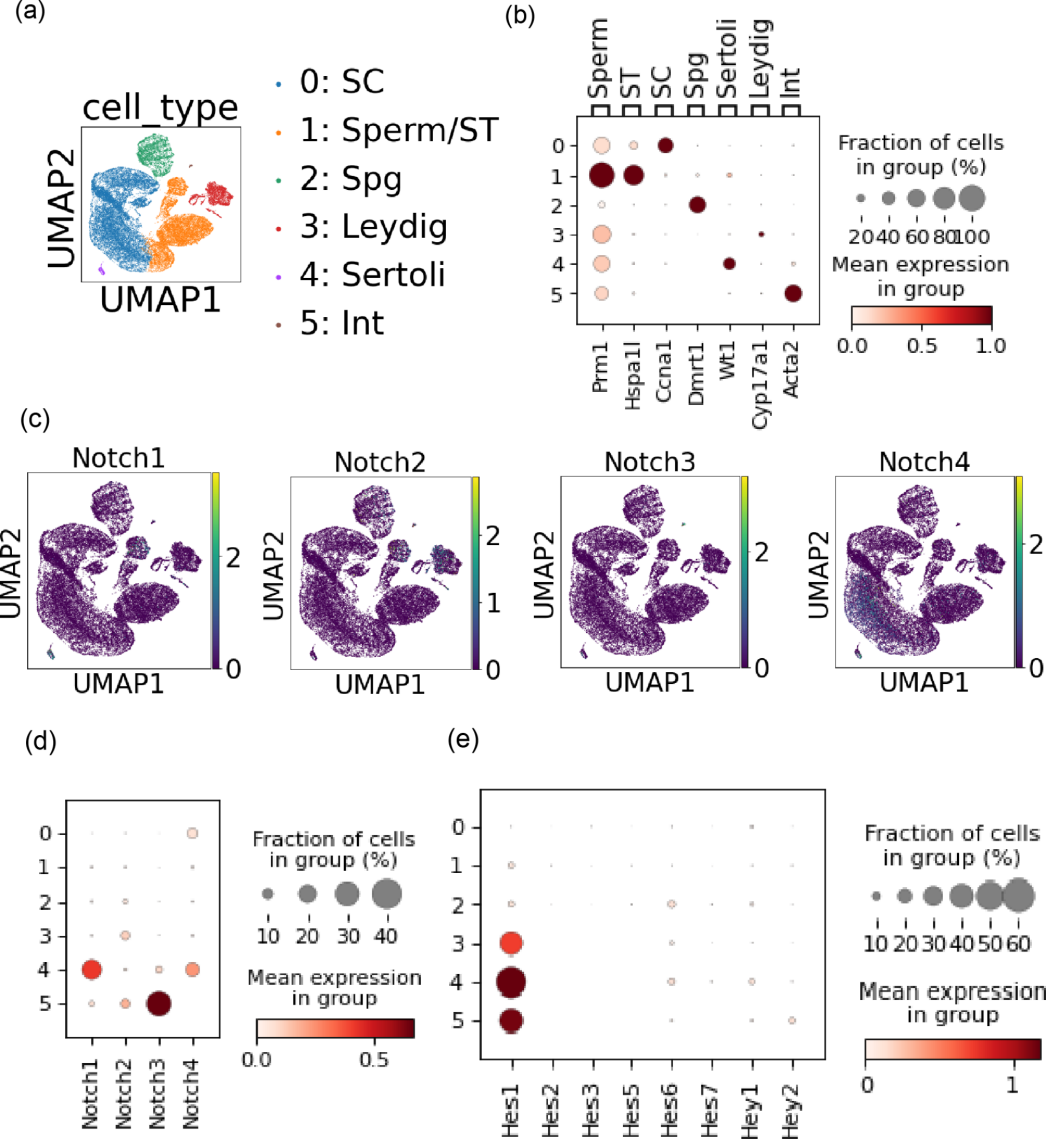
Another mouse testes scRNAseq re-analysis supported lack of *Notch1* expression in the testicular germ cells. (**a**) Six clusters were generated from the mouse testes dataset. (**b**) Characterization of each cluster with indicated marker genes. (**c, d**) The expression levels of the Notch receptors were plotted on the UMAP and dot plot. (**e**) The expression levels of the downstream targets of Notch receptors were plotted.

To validate our experimental and scRNAseq findings, we next carried out an additional round of scRNA-seq analysis using another previously reported mouse testes dataset (GSE112393) [16]. UMAP clustering yielded six clusters characterized with following marker genes: *Prm1* (sperm, cluster #1), *Hspa1l* (ST, cluster #1), *Ccna1* (SC, cluster #0), *Dmrt1* (Spg, cluster #2), *Wt1* (Sertoli, cluster #4), *Cyp17a1* (Leydig, cluster #3), *Acta2* (Int, cluster #5) (Fig. 3a and b). We plotted the expression profiles of Notch receptors on the UMAP and dot plot (Fig. 3c and d). *Notch1 and Notch4* were expressed in cluster #4 (Sertoli), but not in the testicular germ cells (cluster #0 and #1). *Notch3* was expressed in cluster #5 (Int) while *Notch2* was rarely expressed. Among *Hes*/*Hey* family downstream genes, expressed was *Hes1* in cluster #3 (Leydig), #4 (Sertoli) and #5 (Int) (Fig. 3e). This result (GSE112393) was concordant with our previous scRNAseq re-analysis (GSE104556) in that *Notch1* might not be the major receptor in the testicular germ cells.

## Discussion

By using transgenic mouse system and scRNAseq analysis, this study raised a question to a previous report that suggested *Notch1* expression in the male germ cells [10]. In this report, Huang et al. used *Stra8-iCre* mouse to induce gene recombination at early spermatogonial cells (postnatal day3) and suggested that overexpression of Notch1 intracellular domain (aberrant activation of *Notch1* signaling) severely affected sperm development whereas *Notch1* deletion had no apparent effect. Although our result was contradictory to this report that reported Notch1 expression, the main body of the report that gain-of-function, instead of loss-of-function, of *Notch1* abrogated sperm development was natural given the results of our current study because the sperms might develop independently of *Notch1* signaling.

Delta-Notch signaling pathway components are suggested to be involved in gamete formation in many non-mammalian species [6–8]. In *Xenopus laevis*, X-Delta-2 mediates migration of the primordial germ cells [8]. Our scRNAseq re-analyses of the mouse datasets suggested that *Notch1* expressed in Sertoli cells that agreed with a previous study [33] although the testicular germ cells are clearly different from Sertoli cells. In addition, the contribution of *Notch3* in mouse (GSE112393) and human (GSE142585) (See Additional File 2) testicular interstitial cells might reflect the contribution of *Notch3* in the blood vessels since this gene is involved in a hereditary arteriopathy named cerebral autosomal dominant arteriopathy with subcortical infarcts and leukoencephalopathy (CADASIL) [34]. The expression levels of *Notch2* and *Notch4* were relatively low in our scRNAseq re-analyses of four datasets, leaving an uncertainty of their contributions in the testis physiology. Importantly, differentiating testicular germ cells lacked the *Notch1* expression in all four datasets from mouse and human. Therefore, our transgenic mouse experiment suggesting that *Notch1* signaling is lacking in the seminiferous tubules is reasonable.

## Conclusion

*Notch1* signaling is rare in the murine developing sperm and that might partially constitute the reason gain-of-function, instead of loss-of-function, of *Notch1* led to abnormalities in the sperm development in a previous paper by Huang et al. [10].

### Limitations

Since our scRNAsq analysis was carried out only for gonadal cells, our conclusion could not be generalized for gamete formation as a whole, except for *Notch1* signaling in mouse.

## Electronic supplementary material

Below is the link to the electronic supplementary material.

Supplementary Material 1

Supplementary Material 2

## Data Availability

The datasets generated and/or analyzed during the current study are available at our GitHub repositories (https://github.com/Naoto-Sambe/sperm) (https://github.com/MasaharuYoshihara/Sambe_Sperm_Original_Data).

## Abbreviations

BV: Blood and vascular cell
CADASIL: Cerebral autosomal dominant arteriopathy with subcortical infarcts and leukoencephalopathy
EGFP: Enhanced green fluorescent protein
Int: Interstitial cell
macro: Macrophage
ST: Spermatid
SC: Spermatocyte
Spg: Spermatogonial cell
scRNAseq: Single-cell RNA-seq
tdsRed: Tandem dsRed
UAS: Upstream activating sequence
UMAP: Uniform manifold approximation and projection
