# Supplementary material for: Analysis of Notch1 signaling in mammalian sperm development
Source: BMC Res Notes. 2023 Jun 19;16:108. doi: 10.1186/s13104-023-06378-z (PMC10280896; doi:10.1186/s13104-023-06378-z)

Additional File 1

Title of data: Full-length blots/gels of genotyping PCR

Description of data:

Additional File 1 contains the full-length blots/gels of genotyping PCR that is the original image for Figure 1e.

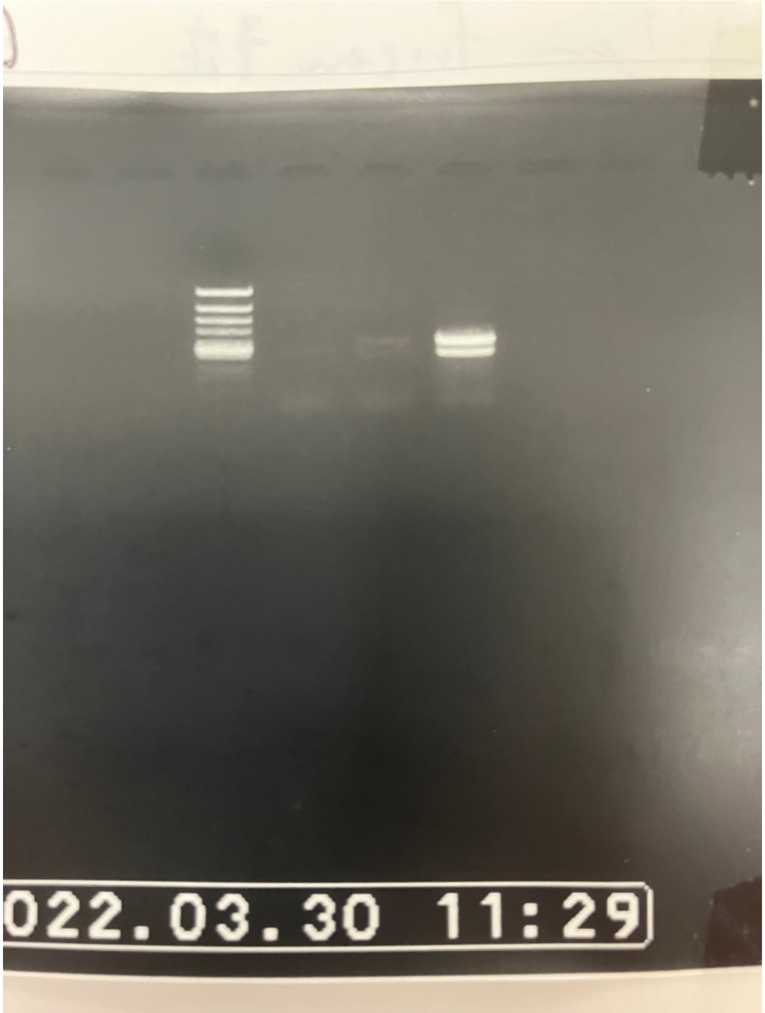

Supplement: Supplementary file 1 — Supplementary Material 1 [file 13104_2023_6378_MOESM1_ESM.pdf]
