# Supplementary material for: Analysis of Notch1 signaling in mammalian sperm development
Source: BMC Res Notes. 2023 Jun 19;16:108. doi: 10.1186/s13104-023-06378-z (PMC10280896; doi:10.1186/s13104-023-06378-z)
Supplement: Supplementary file 2 — Supplementary Material 2 [file 13104_2023_6378_MOESM2_ESM.pdf]

Additional File 2

Title of data: Re-analyses of scRNAseq datasets from human testes

Description of data:

Additional File 2 contains the description of the re-analyses of scRNAseq datasets from human testes (GSE124263 and GSE142585 from human testes) (1) (2) including their methods, results and discussion.

## Introduction

To examine species-difference, the contributions of NOTCH receptors in human were analyzed using two previously reported scRNAseq datasets (GSE124263 and GSE142585 from human testes) (1) (2) from human testes.

## Methods

We downloaded the two datasets of the previously published scRNAseq (GSE124263 and GSE142585 from human testes) (1) (2) from NCBI Gene Expression Omnibus (https://www.ncbi.nlm.nih.gov/geo/). We used Scanpy (v1.7.0) (3) to conduct quality control, dimension reduction with principal component analysis ("scanpy.tl.pca" function)

and UMAP ("scanpy.tl.umap" function) before clustering (Leiden method or Louvain method). Single-cell RNA sequencing analysis was completed in Python 3.6.13 in an Ubuntu 20.04 LTS environment.

For the GSE124263 dataset, we selected and used 4561 cells out of 3065 highly variable genes after removing the doublet cells judged by Scrublet (v0.2.3) (4). For the other human dataset (GSE142585), we used 13628 cells and 6267 highly variable genes after removing the doublet cells judged by Scrublet (v0.2.3) (4). The following marker genes were used: *PRM1* (Sperm) (5), *CCNA1* (spermatocyte, SC) (6), *STK31* (7), *DMRT1* (spermatogonial cell, Spg) (8), *WT1* (Sertoli cell) (9), *CYP17A1* (Leydig cell) (10), *CD68* (macrophage, macro) (11), *ACTA2* (interstitial cell, Int) (12), *CD34* (Blood and vascular cell, BV) (13).

**Results and Discussion**

*A re-analysis of a previously reported scRNAseq dataset from human testes (GSE124263)*

Using GSE124263 datasets, we ran principal component analysis (PCA) and uniform manifold approximation and projection (UMAP) and identified five clusters (Supplementary Figure 1a). Each cluster was characterized with the marker genes as

follows: *ACTA2* (Int, cluster #0), *STK31* (SC, cluster #1), *WT1* (Sertoli, cluster #2), *CD68* (Macro, cluster #3), and *PRM1* (Sperm, cluster #4) (Supplementary Figure 1b). Then, we examined NOTCH receptor expressions on UMAP (Supplementary Figure 1c) and dot plot (Supplementary Figure 1d). As previously reported (14), *NOTCH1* was expressed in cluster #2 (Sertoli) while it was rarely expressed in the germ cells (cluster #0 and #4). *NOTCH4* and *NOTCH2* were expressed in cluster #2 (Sertoli) and #0 (Int), respectively whereas *NOTCH3* was rarely expressed. Among HES/HEY family downstream targets, the enriched expression was observed for *HES1* in cluster #2 (Sertoli) (Supplementary Figure 1e). Since *HES1* was induced by many signaling molecules such as FGF (15), it is uncertain whether *HES1* expression observed here is caused by *NOTCH1* or *NOTCH4* although low *NOTCH1* expression might not favor this notion. Even if *NOTCH1* expression is significant in Sertoli cells, it is important to note that *NOTCH1* expression level was low in the germ cells that agreed with our experimental and mouse scRNAseq examinations.

**Supplementary Figure1**

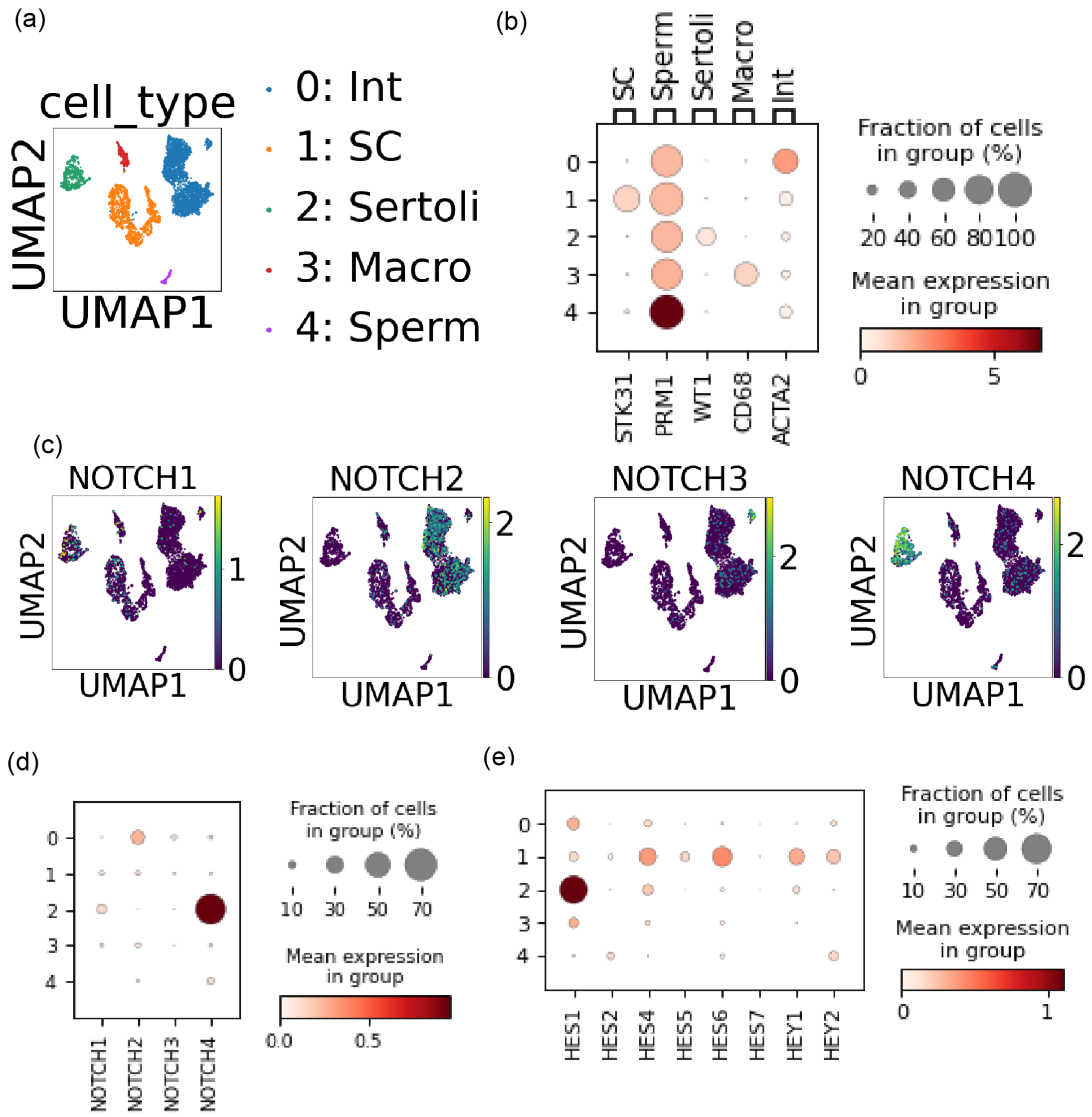

**Supplementary Figure1 Re-analysis of a human testes dataset (GSE124263)**

(a) Five clusters were identifiable in the UMAP. (b) Characterization of each cluster with the indicated genes. (c, d) The expression levels of NOTCH receptors were examined on the UMAP and dot plot. (e) The expression levels of downstream targets of *NOTCH* signaling were plotted.

*Another re-analysis of a previously reported scRNAseq dataset from human testes (GSE142585)*

To validate our result using GSE124263, we also used GSE142585 datasets and ran PCA and UMAP, and identified six clusters (Supplementary Figure 2a). Each cluster was characterized with the marker genes as follows: *CCNA1* (SC, cluster #0), *PRM1* (Sperm, cluster #1), *ACTA2* (Int1, cluster #2), *DMRT1* (Spg, cluster #3), *CD34* (BV, cluster #4), *ACTA2* (Int2, cluster #5) (Supplementary Figure 2b). Sertoli and Leydig cells were hardly identified in this dataset. Then, we examined NOTCH receptor expressions on UMAP (Supplementary Figure 2c) and dot plot (Supplementary Figure 2d). *NOTCH2 and NOTCH3* were expressed in cluster #2 (Int1) and #5 (Int2), respectively. Among HES/HEY family downstream targets, the enriched expression was observed for *HES4* were in cluster #5 (Int2) (Supplementary Figure 2e). Importantly, *NOTCH1* expression

level was low in the germ cells that agreed with our experimental results, and mouse or human scRNAseq examinations.

**Supplementary Figure2**

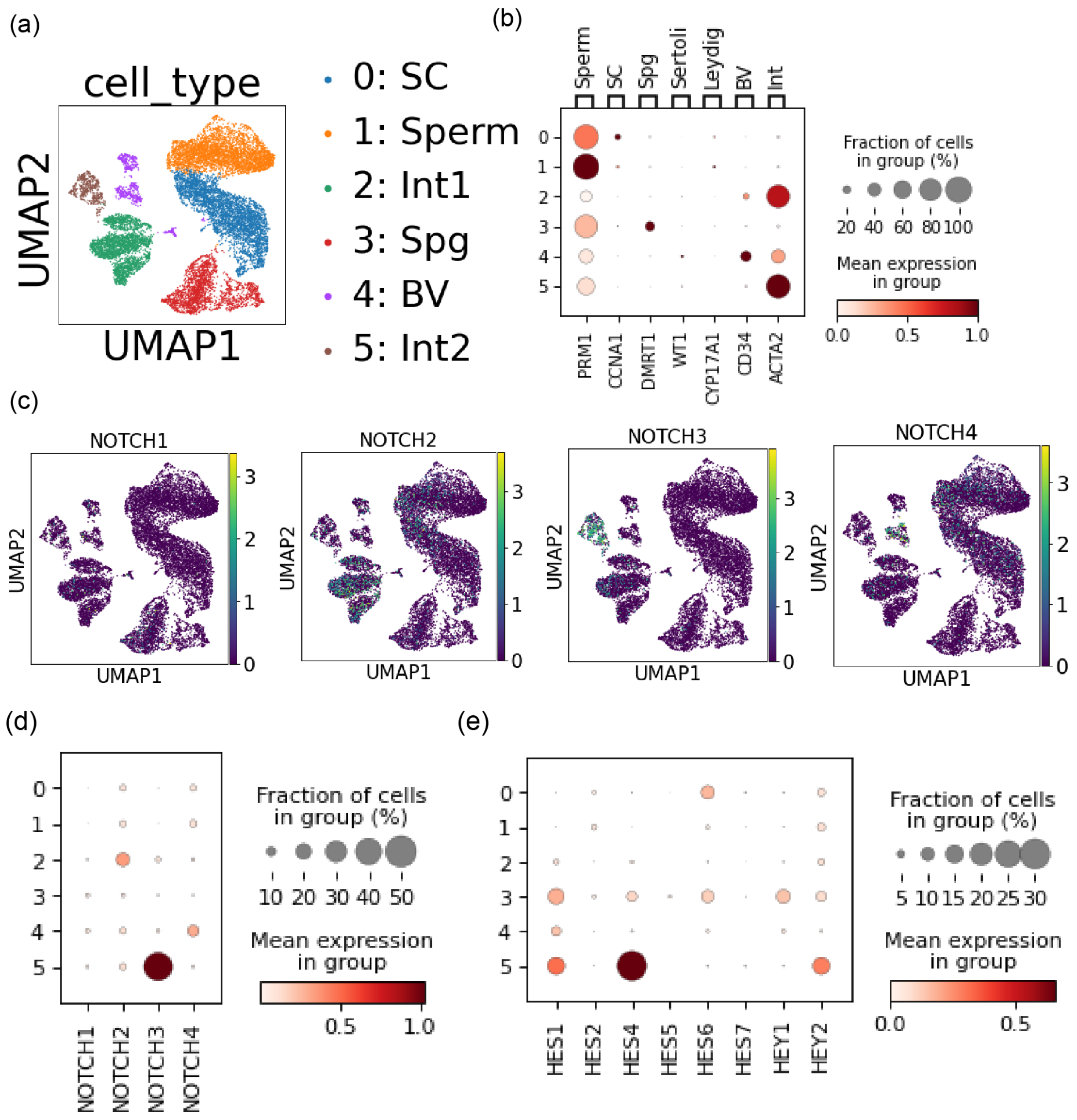

**Supplementary Figure 2 Re-analysis of a human testes dataset (GSE142585)**

(a) Six clusters were identifiable in the UMAP. (b) Characterization of each cluster with the indicated genes. (c, d) The expression levels of NOTCH receptors were examined on the UMAP and dot plot. (e) The expression levels of downstream targets of *NOTCH* signaling were plotted.

## References


1. Sohni A, Tan K, Song HW, Burow D, de Rooij DG, Laurent L et al. The Neonatal and Adult Human Testis Defined at the Single-Cell Level. Cell Rep. 2019;26(6):1501-1517.e4.
2. Shami AN, Zheng X, Munyoki SK, Ma Q, Manske GL, Green CD et al. Single-Cell RNA Sequencing of Human, Macaque, and Mouse Testes Uncovers Conserved and Divergent Features of Mammalian Spermatogenesis. Dev Cell. 2020;54(4):529-547.e12.
3. Wolf FA, Angerer P, Theis FJ. SCANPY: large-scale single-cell gene expression data analysis. Genome Biol. 2018;19(1):15.
4. Wolock SL, Lopez R, Klein AM. Scrublet: Computational Identification of Cell Doublets in Single-Cell Transcriptomic Data. Cell Syst. 2019;8(4):281-291.e9.

5. Ariel M, McCarrey J, Cedar H. Methylation patterns of testis-specific genes. Proc Natl Acad Sci U S A. 1991;88(6):2317-21.

6. Panigrahi SK, Manterola M, Wolgemuth DJ. Meiotic failure in cyclin A1-deficient mouse spermatocytes triggers apoptosis through intrinsic and extrinsic signaling pathways and 14-3-3 proteins. PLoS One. 2017;12(3):e0173926.

7. Zhou J, Leu NA, Eckardt S, McLaughlin KJ, Wang PJ. STK31/TDRD8, a germ cell-specific factor, is dispensable for reproduction in mice. PLoS One. 2014;9(2):e89471.

8. Looijenga LH, Hersmus R, Gillis AJ, Pfundt R, Stoop HJ, van Gurp RJ et al. Genomic and expression profiling of human spermatocytic seminomas: primary spermatocyte as tumorigenic precursor and DMRT1 as candidate chromosome 9 gene. Cancer Res. 2006;66(1):290-302.

9. Wang XN, Li ZS, Ren Y, Jiang T, Wang YQ, Chen M et al. The Wilms tumor gene, Wt1, is critical for mouse spermatogenesis via regulation of sertoli cell polarity and is associated with non-obstructive azoospermia in humans. PLoS Genet. 2013;9(8):e1003645.

10. Laurich VM, Trbovich AM, O'Neill FH, Houk CP, Sluss PM, Payne AH et al. Müllerian inhibiting substance blocks the protein kinase A-induced expression of cytochrome p450 17alpha-hydroxylase/C(17-20) lyase mRNA in a mouse Leydig cell line

independent of cAMP responsive element binding protein phosphorylation. Endocrinology. 2002;143(9):3351-60.

11. Chistiakov DA, Killingsworth MC, Myasoedova VA, Orekhov AN, Bobryshev YV. CD68/macrosialin: not just a histochemical marker. Lab Invest. 2017;97(1):4-13.

12. Wang J, Zohar R, McCulloch CA. Multiple roles of alpha-smooth muscle actin in mechanotransduction. Exp Cell Res. 2006;312(3):205-14.

13. Lai JH, Zhou YJ, Bin D, Qiangchen, Wang SY. Clinical significance of detecting lymphatic and blood vessel invasion in stage II colon cancer using markers D2-40 and CD34 in combination. Asian Pac J Cancer Prev. 2014;15(3):1363-7.

14. Hasegawa K, Okamura Y, Saga Y. Notch signaling in Sertoli cells regulates cyclical gene expression of Hes1 but is dispensable for mouse spermatogenesis. Mol Cell Biol. 2012;32(1):206-15.

15. Nakayama K, Satoh T, Igari A, Kageyama R, Nishida E. FGF induces oscillations of Hes1 expression and Ras/ERK activation. Curr Biol. 2008;18(8):R332-4.
